# Expedient synthesis of 1,6-anhydro-α-D-galactofuranose, a useful intermediate for glycobiological tools

**DOI:** 10.3762/bjoc.10.172

**Published:** 2014-07-21

**Authors:** Luciana Baldoni, Carla Marino

**Affiliations:** 1 CIHIDECAR-CONICET-UBA, Departamento de Química Orgánica, Facultad de Ciencias Exactas y Naturales, Universidad de Buenos Aires, Pabellón II, Ciudad Universitaria, 1428 Buenos Aires, Argentina, Tel/Fax: +54-11-45763352

**Keywords:** 1,6-anhydro-α-D-Gal*f*, galactofuranosyl iodide, galactofuranosyl precursor, per-*tert*-butyldimethylsilyl-β-D-galactofuranose

## Abstract

A new and efficient three-step procedure for the synthesis of 1,6-anhydro-α-D-galactofuranose is described. The key step involves the formation of the galactofuranosyl iodide by treatment of per-*O*-TBS-D-Gal*f* with TMSI, the selective 6-*O*-desilylation by an excess of TMSI, and the simultaneous nucleophilic attack of the 6-hydroxy group on the anomeric carbon, with the iodide as a good leaving group. This compound is a good precursor for building blocks for the construction of 1→6 linkages.

## Introduction

Anhydro sugars are formed by the intramolecular elimination of a water molecule, with the simultaneous formation of a new heterocyclic ring of different size. They are valuable intermediates not only in carbohydrate synthesis, but also as starting materials for other natural and non-natural complex products and bioactive compounds. Among the glycosans, the anhydro sugars involving the anomeric center in the ring formation, the 1,6-anhydro sugars are the most common and useful building blocks [1–2]. They can play a role in synthetic methodologies aiming at the obtainment of regioselectively functionalized sugars in a few steps, which could give easy access to convenient glycosyl donors and acceptors [3].

**Figure 1:**
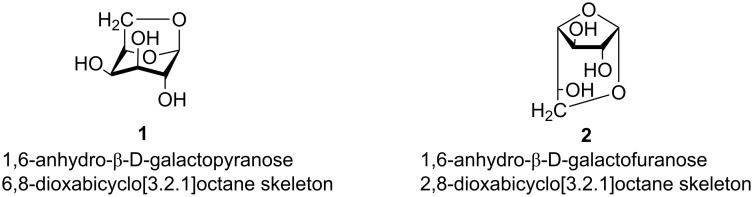
Possible 1,6-anydro derivatives for D-galactose.

Some sugars, for example galactose, can afford not only the pyranosic derivative **1** but also the furanosic 1,6-anhydro derivative **2**, both of which may be equipped with [3.2.1] bicyclic skeletons (Figure 1) [1].

A variety of chemical approaches for the synthesis of 1,6-anhydro sugars have been developed [3–10]. Two classes of methods for the synthesis of **2** can be discriminated, the first of which starts from free galactose (D-Gal) and afford mixtures of **1** and **2** and the second starts from a galactofuranose (D-Gal*f*) template conveniently derivatized. Pioneer procedures for the synthesis of **2** involved the pyrolysis of D-Gal under reduced pressure [11–12] and the acid treatment under heating [13], with the subsequent tedious separation from several byproducts, including the pyranosic analogue **1**. Compound **2** was thus obtained in very low yield. More recently, **2** was obtained in 32% yield by heating with a resin as an acid catalyst. Despite the greater smoothness of the method, byproducts were also formed, rendering the purification difficult [14].

Compound **2** obtained by these procedures was used to afford polymers to explore their possible applications in the field of biochemistry and pharmacology, as their properties differ from those of the corresponding monosaccharides, and they have a high density of functional groups that can be modified to obtain novel materials [14–15]. Benzylated **2** was polymerized under cationic conditions, which afforded a material not completely characterized, presumably formed by β-D-Gal*f* units [15]. Free compound **2**, as well as the D-Man*f* and D-Glc*f* analogues, was also polymerized under cationic conditions to yield a hyperbranched polysaccharide with α- and β-linked pyranosidic and furanosidic units [14].

**Scheme 1:**
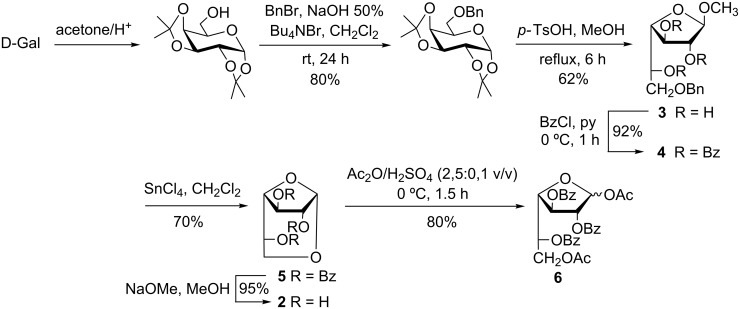
Reported synthesis of 1,6-anhydro-α-D-galactofuranose [16–17].

On the other hand, when compound **2** was envisioned as a D-Gal*f* template, the synthesis was devised starting from convenient derivatives of D-Gal*f* in order to avoid the presence of **1**. For example, compound **2** was synthesized in the past in six steps from galactose (Scheme 1) [16]. The 1,6-ring closure was produced by the *O*-debenzylation of the 6-hydroxy group of **4** and the nucleophilic attack of this hydroxy group to C-1, promoted by SnCl_4_. An optimized synthesis of **2** following this strategy has recently been described with an overall yield of 48% comprising several column chromatography purification steps [17].

**Figure 2:**
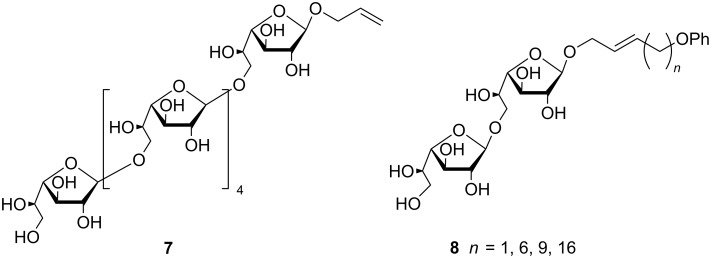
Examples of glycobiological tools synthesized from compound **5** [29–31].

The essential role of galactofuranose in the antigenic response of various pathogenic microorganisms [18–20] has triggered the interest for the development of synthetic methods for D-Gal*f* precursors and efficient galactofuranosylation methods [21–25]. D-Gal*f* units have been shown to be *O*-glycosidically linked to other D-Gal*f* units by 1→6 linkages in many natural structures, e.g., in pathogenic *Mycobateria* and *Aspergillius* spp and others [25–28]. Benzoylated compound **5** is a good precursor of D-Gal*f* derivatives with differentially protected hydroxy groups at position 1 and 6, for example the diacetyl derivative **6** obtained by the acetolysis of **5** (Scheme 1) [16]. In this way, compound **5** would give access to donors in which the 6-position could subsequently be manipulated for the construction of a 1→6 linkage. Based on this strategy, Ning and co-workers synthesized the β-(1→6)-linked hexasaccharide **7** [29], and Kiessling‘s group developed the synthesis of compounds **8**^.^used for the characterization of GlfT2, one of the two galactofuranosyl transferases involved in the biosynthesis of D-Gal*f*-containing molecules (Figure 2) [30–31].

Our laboratory has long been involved in the development of new galactofuranosyl derivatives and galactofuranosylation methodologies [32]. In this context, we herein report on an efficient three-step synthesis of 1,6-anhydro-α-D-galactofuranose (**2**) from per-*O*-TBS-β-D-galactofuranose (**9**) as a more efficient alternative to existing methods.

## Results and Discussion

**Scheme 2:**
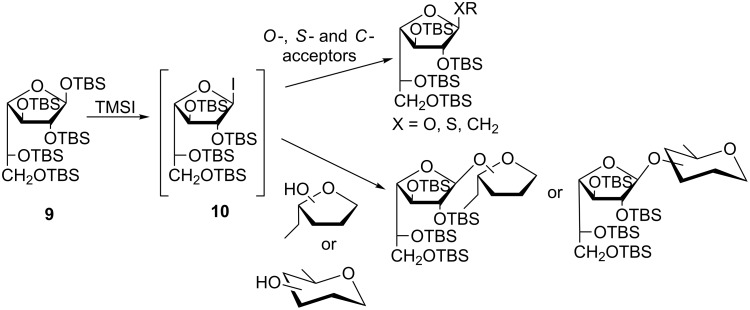
D-Galactofuranosylation by the glycosyl iodide method [32–35].

In the framework of our project for the development of galactofuranosyl derivatives and glycosylation methods, we have reported the synthesis of per-*O*-TBS-β-D-galactofuranose (**9**), a convenient precursor of D-Gal*f* units, and its glycosylation via the in situ generation of galactofuranosyl iodide **10** (Scheme 2) [32–35]. Galactofuranosyl iodides were not previously described, and **10** proved to be useful for the synthesis of several D-Gal*f*-containing molecules (Scheme 2) [32].

The reported procedure consisted in the treatment of compound **9** with 1.2 equiv of TMSI in anhydrous CH_2_Cl_2_ at 0 ºC until the total conversion of **7** into two lower moving products was observed by TLC: the 1-iodo intermediate **10** (*R*_f_ = 0.70, 10:1 hexane/EtOAc) and 2,3,5,6-tetra-*O*-TBS-α,β-D-galactofuranose (*R*_f_ = 0.54) formed as a result of the hydrolysis of **10** on the silica gel plate. The addition of simple alcohols or partially protected sugars as acceptors and EtN(iPr)_2_ as an acid scavenger led to the complete consumption of both compounds and afforded the corresponding glycosides (Scheme 2) [32]. With an excess of TMSI, a third product (*R*_f_ = 0.62) was formed, which was not consumed during the reaction and was still present in the product mixture after the work-up. The ^1^H NMR spectrum of this product showed a doublet at δ 5.06 with a relatively large *J*_1,2_ value (4.5 Hz). This signal correlated with a signal at 98.4 ppm in the ^13^C NMR spectrum, both indicative of the α-configuration. Signals corresponding to C-5 and C-6 showed similar chemical shifts. The signal corresponding to C-6 (δ 65.9) was shifted slightly downfield compared to the same signal in compound **9** (64.7 ppm), while the signal corresponding to C-5 (64.2 ppm) was significantly deshielded (10 ppm) with respect to C-5 of compound **9**. No aglycone signals were observed. In order to elucidate the structure of this compound we *O*-desilylated it by treatment with *n-*Bu_4_NF (TBAF) in THF [36]. The product obtained (96%) was faster moving than galactose on TLC (*R_f_* = 0.60, 7:1:2 *n-*PrOH/NH_3_/H_2_O) and showed ^1^H and ^13^C NMR spectra coincident with the data reported for 1,6-anhydro-α-D-galactofuranose (**2**) [16,37]. With the objective of optimizing the conditions for glycosylations via iodide **10**, the formation of **12** was suppressed by strict control of the TMSI amount employed.

**Scheme 3:**
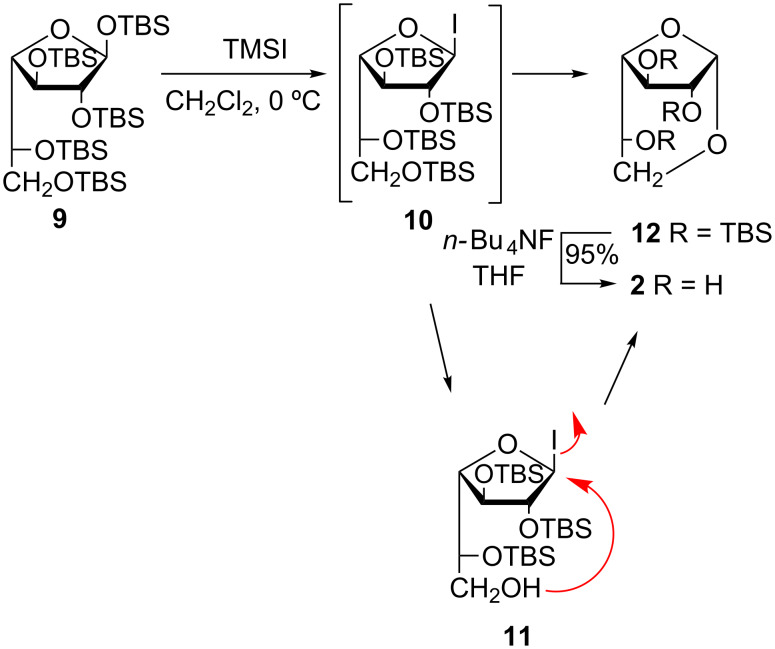
Synthesis of 1,6-anhydro-α-D-galactofuranose from per-*O*-*tert*-butyldimethylsilyl-D-Gal*f*.

Taking into consideration how easily compound **12** was obtained and the versatility of anhydro sugars as intermediates for the preparation of biologically important oligosaccharides [3], we decided to investigate the conditions to obtain it as a main product. We reasoned that during the treatment of **9** with TMSI, in addition to the formation of the anomeric iodide, the 6-hydroxy group could be desilylated by the acid medium developed during the iodide formation (**10** → **11**). Then, the free 6-hydroxy group could carry out an intramolecular attack of the anomeric carbon, with iodide as a good leaving group, affording the 1,6-anhydro derivative **12** (Scheme 3).

By treatment of **9** with an excess of TMSI (2.25 equiv) in CH_2_Cl_2_ at room temperature for 5 h, compound **12** was obtained as a single product in 65% yield. Conducting the reaction at low temperature instead (−20 °C), a lower moving product was detected, presumable **11**, which could not be isolated. The use of molecular sieves, which improve the reaction of **10** with alcohols or complex acceptors [32–34], should be avoided in this case as it slows down the reaction. Based on monitoring the reaction by ^1^H NMR (CDCl_3_) spectroscopy we observed that the anomeric signal of **9** (5.15 ppm, *J*_1,2_ 2.6 Hz) was slowly transformed into a broad singlet at 6.53 ppm, corresponding to H-1 of **10** [32], followed by the transformation into the anomeric signal of **12** (5.06 ppm, *J*_1,2_ 4.5). However, the deprotection of the 6-hydroxy group of galactofuranosides affects the pattern of H-6 and H-6’ in the ^1^H NMR spectrum, effectively equalizing them, as was shown before [38]. During the reaction, the pair of double-doublets of H-6 and H-6’ (3.68 and 3.54 ppm) of compound **9** [32] were transformed in a double-doublet (3.72 ppm) and an apparent triplet (3.60 ppm), corresponding to the H-6 and H-6’ of **12** [32]. In between these two signals an intense doublet corresponding to equivalent H-6,6´ (3.58 ppm) of **11** was observed, in accordance with the behavior of other free HO-galactofuranosides [38], which supports the intermediate formation of **11**.

The treatment of **9** with SnCl_4_ afforded compound **12**, but in a lower yield due to the *O*-desilylation of another hydroxy group. The addition of BF_3_·OEt_2_ to recently formed **10** resulted in the formation of compound **12**.

**Figure 3:**
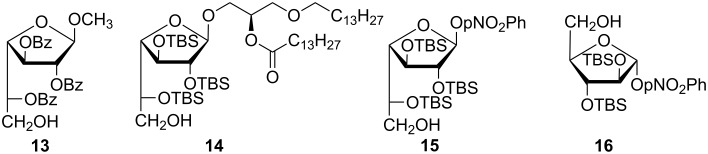
Furanosic derivatives with free primary hydroxy group.

Several factors favor the formation of the bicyclic system of compound **12**, such as the galactose structure itself, the presence of a good leaving group at C-1, and the electron-donating nature of the TBS groups. Thus, while compounds **13** [38] and **14** [39] were prepared by treatment with Lewis acids of fully protected precursors and proved to be stable and therefore useful as synthetic intermediates, attempts to prepare compound **15** by treatment with BF_3_·OEt_2_ of the persilylated precursor failed and inevitably led to the anhydro derivative **12**. Moreover, whereas treatment with TFA/THF/H_2_O 90:5:2.5 of 4-nitrophenyl per-*O*-TBS-α-D-Ara*f* afforded **16**, 4-nitrophenyl per-*O*-TBS-β-D-galactofuranoside did not lead to compound **15** under the same conditions and compound **12** was obtained instead (Figure 3).

The *O*-desilylation of **12** was performed by treatment with *n-*Bu_4_F as previously optimized [32–34], affording compound **2** in almost quantitative yield (Scheme 3).

## Conclusion

In conclusion, we have described a new and concise procedure for the synthesis of the 1,6-anhydro derivatives **2** and **12**, the key step of which proceeds by a cascade set of three consecutive reactions. The method compares well to existing methods and by avoiding cumbersome steps, such as a benzylation and several column chromatography purifications, is an effective approach. Compounds **2** and **12** represent profitable intermediates to easily access donors and acceptors for the synthesis of Gal*f*-containing molecules as biochemical tools.

## Experimental

### General methods

Analytical thin-layer chromatography (TLC) were performed on Silica Gel 60 F_254_ (Merck) aluminum supported plates (layer thickness 0.2 mm) with solvent systems given in the text. Visualization of the spots was effected by exposure to UV light and charring with a solution of 10% (v/v) sulfuric acid in EtOH containing 0.5% *p*-anisaldehyde. Column chromatography was carried out with Silica Gel 60 (230–400 mesh, Merck). Optical rotations were measured with a Perkin-Elmer 343 digital polarimeter. Nuclear magnetic resonance (NMR) spectra were recorded with a Bruker AMX 500 spectrometer. Assignments of ^1^H and ^13^C were assisted by 2D ^1^H COSY and HSQC experiments. High resolution mass spectra (HRMS–ESI^+^) were recorded in a Bruker micrOTOF-Q II spectrometer.

**2,3,5-Tri-*O*-*tert*-butyldimethylsilyl-1,6-anhydro-α-D-galactofuranose (12).** A solution of **9** [32] (0.90 g, 1.20 mmol) in anhydrous CH_2_Cl_2_ (15 mL) was cooled to 0 ºC and stirred for 10 min under Ar. Then, iodotrimethylsilane (2.25 equiv, 0.38 mL, 2.70 mmol) was slowly added by using a syringe (10 min) while stirring was continued at 0 ºC. The reaction was allowed to reach room temperature (18–25 °C) and stirred until TLC monitoring showed the complete transformation of **9**, first in two products with *R*_f_ = 0.70 and 0.54 (10:1 hexane-EtOAc), then the transformation of both products in one with *R*_f_ = 0.62 (5 h). The solution was diluted with CH_2_Cl_2_ (250 mL), washed with NaHCO_3_ (ss) and water, dried (Na_2_SO_4_), and concentrated. The residue was purified by column chromatography (98.6:1.4 → 2:1, hexane–EtOAc) affording compound **12** as an amorphous solid (0.272 g, 65%). The analytical data of **12** were identical with those described in ref. [32]: [α]_D_ +42 (*c* 1, CHCl_3_); ^1^H NMR (500 MHz, CDCl_3_) δ 5.05 (d, *J* = 4.5 Hz, 1H, H-1), 4.20 (d, *J* = 1.8 Hz, 1H, H-3), 4.15 (dd, *J* = 1.8, 4.5 Hz, 1H, H-2), 3.97 (ddd, *J* = 4.3, 6.3, 10.5 Hz, 1H, H-5), 3.91 (broad d, *J* = 4.0 Hz, 1H, H-4), 3.72 (ddd, *J* = 1.5, 6.2, 10.5 Hz, 1H, H-6), 3.60 (apparent t, *J* = 10.7 Hz, 1H, H-6′), 0.94–0.86 (SiC(C*H*_3_)_3_), 0.12–0.04 (Si(C*H*_3_)_2_); ^13^C NMR (125,8 MHz, CDCl_3_) δ 98.4 (C-1), 85.3 (C-4), 83.2 (C-2), 77.6 (C-3), 65.9 (C-6), 64.2 (C-5), 25.84, 25.89, 25.6 (SiC(*C*H_3_)_3_), 17.9 (Si*C*(CH_3_)_3_), −4.49, −4.57, −4.64, −4.68, −4.93, −5.02 (Si(CH_3_)_2_); Anal. calcd for C_24_H_52_O_5_Si_3_: C, 57.09; H, 10.38; found: C, 56.90; H, 10.52.

**1,6-Anhydro-α-D-galactofuranose (2).** To a solution of **12** (0.12 g, 0.22 mmol) in freshly distilled THF (7 mL), cooled at 0 °C, (*n-*Bu)_4_NF (12 equiv, 2.32 g, 8.88 mmol) was added [36]. The solution was allowed to reach room temperature and then stirring was continued for 3 h until TLC monitoring showed the complete consumption of the starting material. The solution was diluted with water (50 mL), extracted with CH_2_Cl_2_ (2 × 30 mL), and the aqueous phase was concentrated under vacuum. Purification of the residue by column chromatography (20:1 AcOEt/hexane) afforded **2** (0.036 g, 95%), [α]_D_ +54 (*c* 1.0, H_2_O), lit. [16] [α]_D_ +54; ^1^H NMR (500 MHz, D_2_O) δ 5.31 (d, *J* = 4.6 Hz, 1H, H-1), 4.26–4.23 (m, 2H, H-2, H-3), 4.19 (broad d, *J* = 4.2 Hz, 1H, H-4), 4.08–3.99 (m, 2H, H-5, H-6), 3.55 (apparent t, *J* = 10.4 Hz, 1H, H-6′); ^13^C NMR (125.8 MHz, D_2_O) δ 98.6 (C-1), 85.2 (C-4), 80.9 (C-2), 75.4 (C-3), 65.6 (C-6), 62.7 (C-5).

## Supporting Information

File 1 ^1^H and ^13^C NMR spectra of compounds **2** and **12**.
