# Supplementary material for: Expedient synthesis of 1,6-anhydro-α-D-galactofuranose, a useful intermediate for glycobiological tools
Source: Beilstein J Org Chem. 2014 Jul 21;10:1651–6. doi: 10.3762/bjoc.10.172 (PMC4143087; doi:10.3762/bjoc.10.172)
Supplement: File 1 — 1H and 13C NMR spectra of compounds 2 and 12. [file Beilstein_J_Org_Chem-10-1651-s001.pdf]

Supporting Information

for

Expedient synthesis of 1,6-anhydro-α-D-galactofuranose, a useful intermediate for glycobiological tools

Luciana Baldoni and Carla Marino*

Address: CIHIDECAR-CONICET-UBA, Departamento de Química Orgánica, Facultad de Ciencias Exactas y Naturales, Universidad de Buenos Aires, Pabellón II, Ciudad Universitaria, 1428 Buenos Aires, Argentina, Tel/Fax: +54-11-45763352

E-mail: Carla Marino - cmarino@qo.fcen.uba.ar

*Corresponding author

^{1}H and ^{13}C NMR spectra of compounds 2 and 12

Content

^{1}H NMR spectrum of compound **12** (500 MHz, $CDCl_3$)……..……………… S3

^{13}C NMR spectrum of compound **12** (125.8 MHz, $CDCl_3$)…………………... S4

^{1}H NMR spectrum of compound **2** (500 MHz, D_2O)………………………… S5

^{13}C NMR spectrum of compound **2** (125.8 MHz, D_2O)……………………… S6

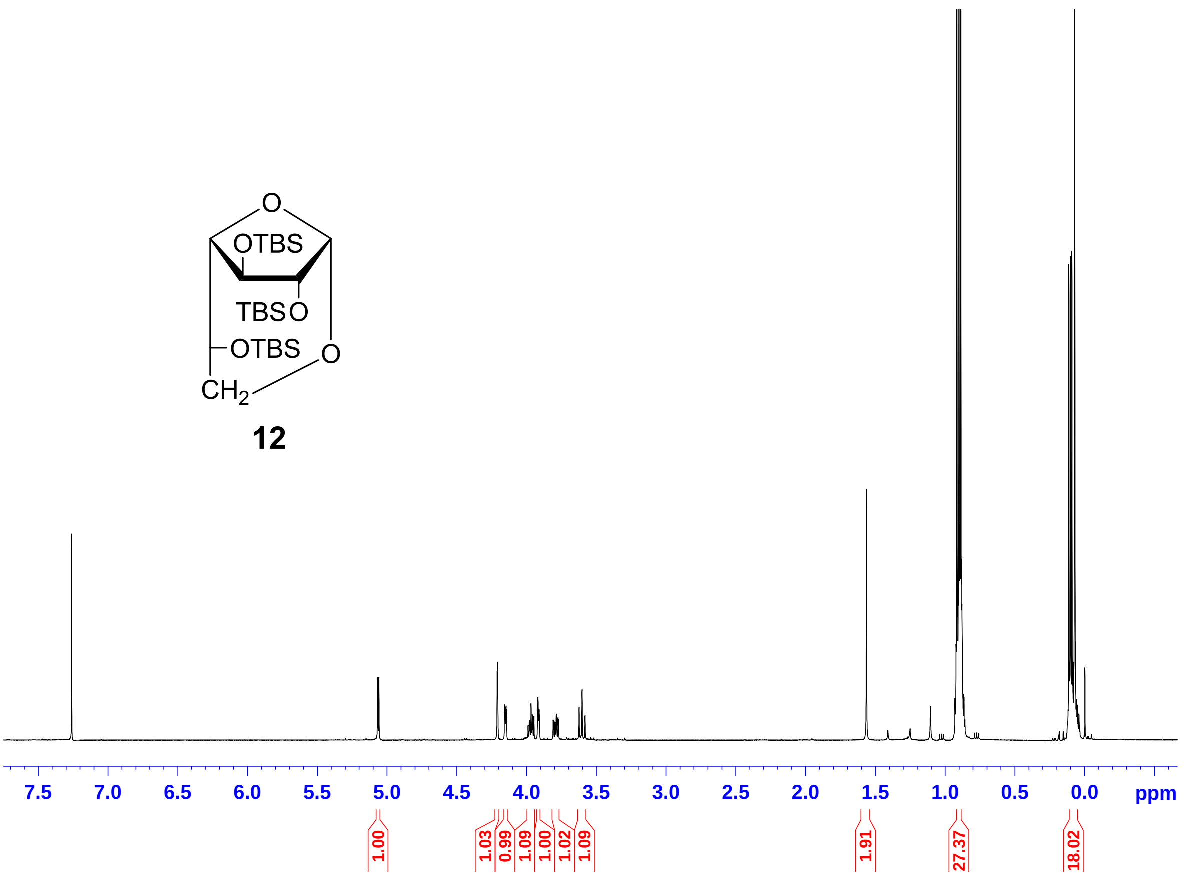
O
OTBS
TBSO
OTBS
O
CH2
12
7.5 7.0 6.5 6.0 5.5 5.0 4.5 4.0 3.5 3.0 2.5 2.0 1.5 1.0 0.5 0.0 ppm
1.00
1.03 0.99 1.09 1.00 1.02 1.09
1.91
27.37
18.02

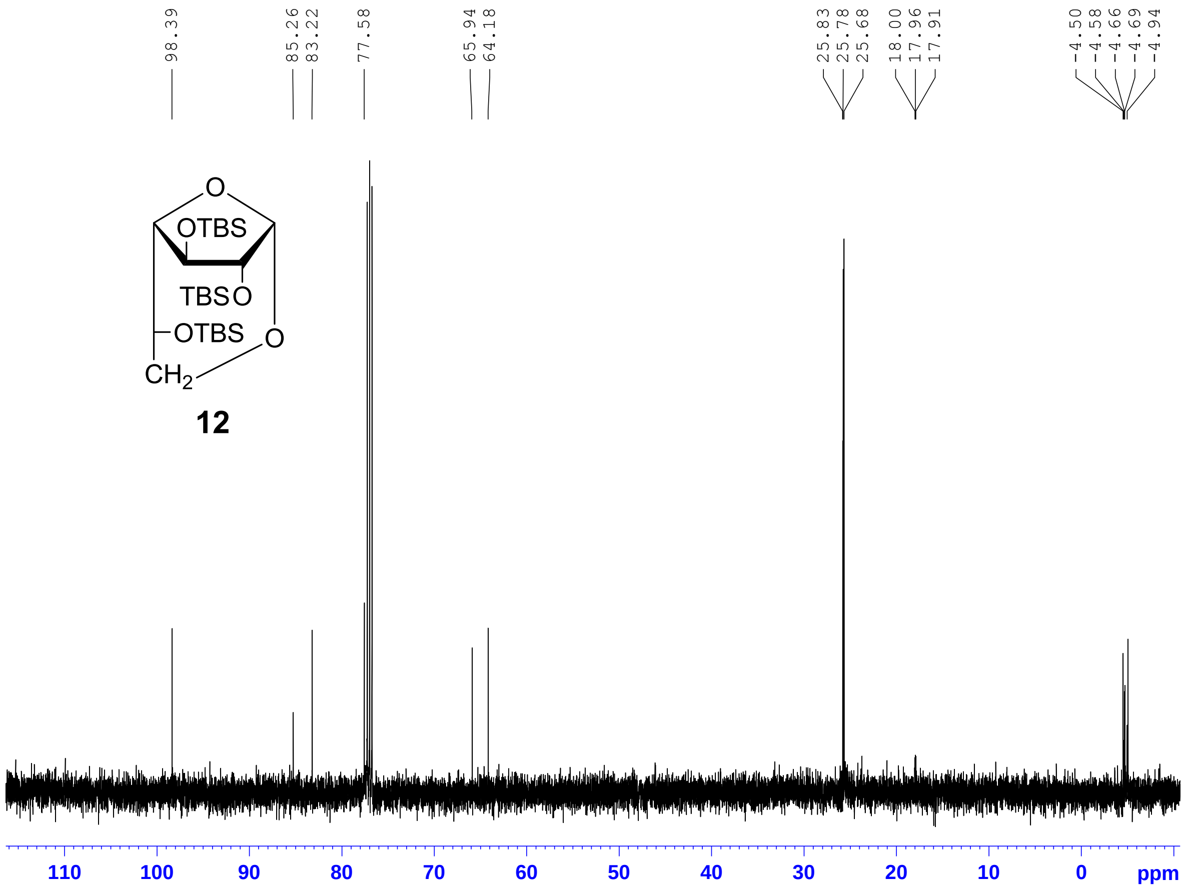
98.39
85.26
83.22
77.58
65.94
64.18
25.83
25.78
25.68
18.00
17.96
17.91
-4.50
-4.58
-4.66
-4.69
-4.94
O
OTBS
TBSO
OTBS
O
CH2
12
110
100
90
80
70
60
50
40
30
20
10
0
ppm

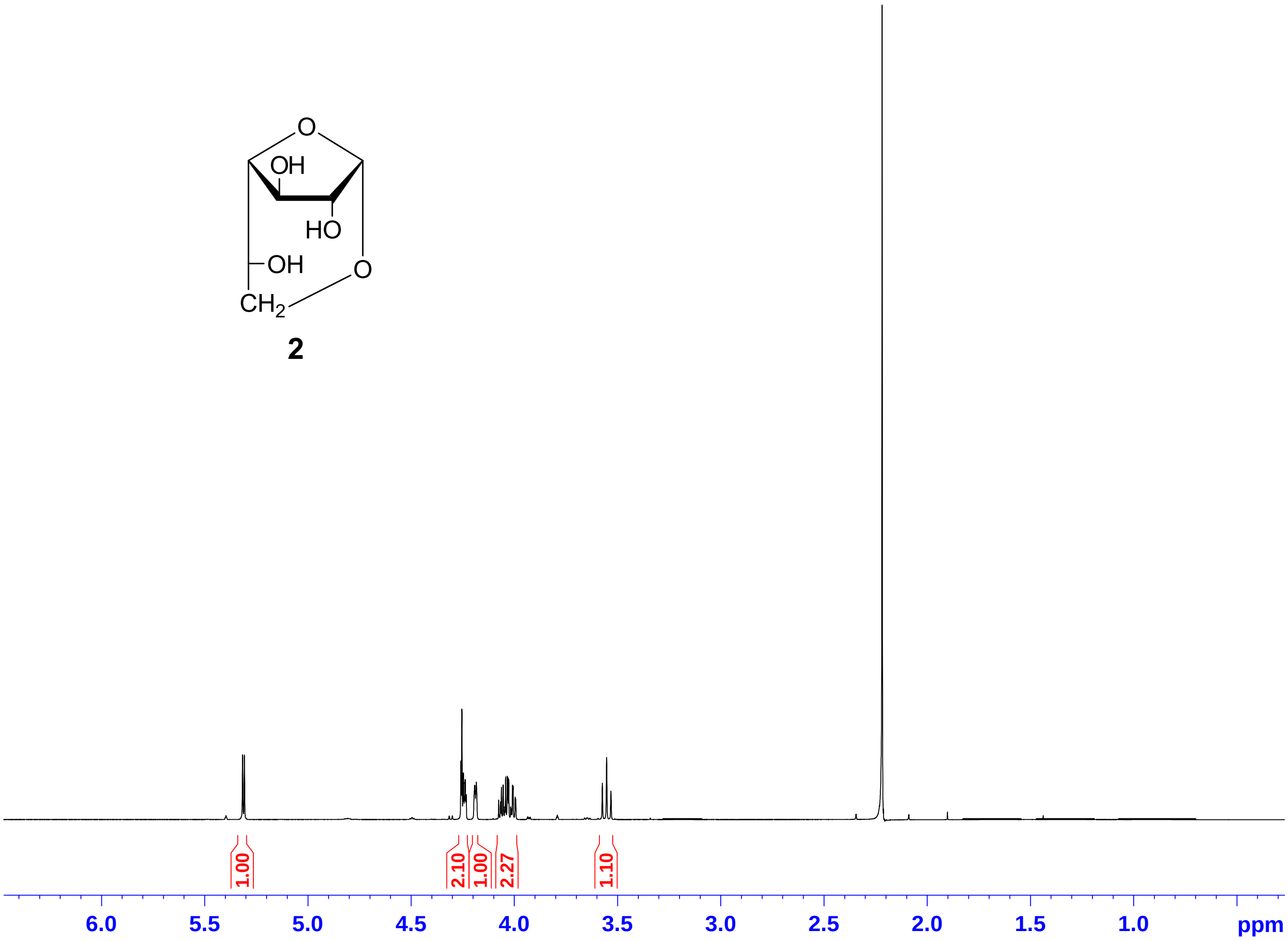
O
OH
HO
OH
O
CH2
2
1.00
2.10
1.00
2.27
1.10
6.0
5.5
5.0
4.5
4.0
3.5
3.0
2.5
2.0
1.5
1.0
ppm

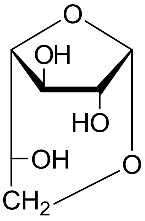
O
OH
HO
OH
O
CH2

2

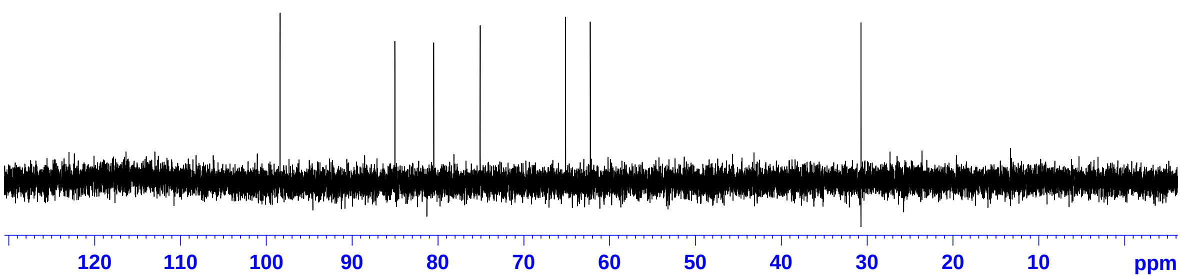
98.61
85.20
80.73
75.27
65.33
62.60
120
110
100
90
80
70
60
50
40
30
20
10
ppm
